# Ultrasound-guided oblique approach for peripheral venous access in a phantom model

**DOI:** 10.1186/2036-7902-4-14

**Published:** 2012-06-15

**Authors:** Heather M Tassone, Vivek S Tayal, Anthony J Weekes, Craymon L Garner, James H Norton

**Affiliations:** 1 Department of Emergency Medicine, Carolinas Medical Center Main, Charlotte, NC 28232, USA; 2 Current address: Department of Emergency Medicine, Loma Linda Medical Center, Loma Linda, CA 92350, USA; 3 Department of Biostatistics, Carolinas Medical Center Main, Charlotte, NC 28232, USA

**Keywords:** Ultrasound, Oblique, Emergency, Peripheral, Vascular, Access, Aspiration

## Abstract

**Background:**

Ultrasound (US) vascular guidance is traditionally performed in transverse (T) and longitudinal (L) axes, each with drawbacks. We hypothesized that the introduction of a novel oblique (O) approach would improve the success of US-guided peripheral venous access. We examined emergency physician (EP) performance using the O approach in a gel US phantom.

**Methods:**

In a prospective, case control study, EPs were enrolled from four levels of physician experience including postgraduate years one to three (PGY1, PGY2, PGY3) and attending physicians. After a brief training session, each participant attempted vessel aspiration using a linear probe in T, L, and O axes on a gel US phantom. Time to aspiration and number of attempts to aspiration were recorded. The approach order was randomized, and descriptive statistics were used.

**Results:**

Twenty-four physicians participated. The first-attempt success rate was lower for O, 45.83%, versus 70.83% for T (*p* = 0.03) and 83.33% for L (*p* = 0.01). The average time to aspiration was 12.5 s (O) compared with 9.47 s (T) and 9.74 s (L), respectively. There were no significant differences between all four groups in regard to total amount of time and number of aspiration attempts; however, a trend appeared revealing that PGY3 and attending physicians tended to aspirate in less time and by fewer attempts in all three orientations when compared with the PGY2 and PGY1 physicians.

**Conclusion:**

In this pilot study, US-guided simulated peripheral venous access using a phantom gel model in a mixed user group showed that the novel oblique approach was not initially more successful versus T and L techniques.

## Background

Peripheral venous access is commonly employed in the emergency department (ED) to obtain blood samples and to administer intravenous medications and fluids. In certain patient populations, the ability to access a vessel is often limited by the ability to locate the vessel by blind aspiration. With increasing frequency, ED physicians have sought to use ultrasound guidance to obtain peripheral lines [1-6].

Traditionally, a transverse or longitudinal sonographic view is used to access a vessel. Each of these approaches has unique advantages and disadvantages. The transverse view allows for the target vessel and its surrounding structures to be seen simultaneously on the screen; however, the needle tip can be lost from view, and posterior vessel penetration can occur without being detected [7-9]. The longitudinal view allows direct real-time visualization of the needle tip and shaft entering the tissue from the skin to the vessel without transducer movement. However, the surrounding structures cannot be simultaneously visualized, and it is easy to ‘slide off’ the structure.

In 2009, Phelan and Hagerty described the use of the oblique technique as an alternative to the transverse and longitudinal approaches for ultrasound-guided central line placement [9]. The oblique approach is a cross-angle approach between the transverse and longitudinal approaches, and we felt it may offer the ‘best of both worlds’ in terms of visualization.

In this study, we hypothesized that the introduction of the oblique technique would improve the success of simulated peripheral venous access compared to traditional approaches by reducing the total number of attempts, reducing time to successful aspiration, and improving first-attempt success rates on a phantom gel model simulating peripheral veins in a cohort of ultrasound-trained emergency physicians of varying experience levels.

## Methods

### Study design and setting

This was a prospective study comparing ultrasound-guided peripheral intravenous access in an out-of-plane *oblique* approach versus the more commonly used out-of-plane *transverse* and in-plane *longitudinal* approaches. The study was conducted in an urban regional emergency department with a total of 100,000 annual visits per year with an emergency medicine residency, emergency ultrasound program of greater than 10 years in duration, and an emergency ultrasound fellowship. A total of 24 emergency medicine physicians enrolled in the study.

### Inclusion criteria

Emergency department attending and resident physicians who were willing to volunteer for the study were eligible for enrollment.

### Exclusion criteria

The faculty and fellows of the Department of Emergency Medicine, Division of Emergency Ultrasound were excluded.

### Protocol

Twenty-four physicians were enrolled into the study from four levels of physician experience including postgraduate year (PGY) one, two, and three and attending physicians. Six physicians were recruited for each of the four groups for a total of 24 participants. The institutional IRB approved the waiver of informed consent for this study.

Each participant attempted vessel aspiration on the same Blue Phantom Ultrasound Training Model Select Series 2 Vessel Original phantom (part number BP0100; Blue Phantom, Redmond, WA, USA). An Ultrasonix Sonix Touch (Vancouver, British Columbia) machine using a L14-5 (38) linear probe, with a frequency of 6 to 14 MHz, was used for each enrollment.

All participants used a 5-mL syringe with a 21-G needle for aspiration. The same vessel in the ultrasound phantom was aspirated by all 24 participants, as the access to the other simulated vessel was blocked with tape. Each participant approached vessel aspiration using the transverse, longitudinal, and oblique approaches in the order instructed by the research assistant. The physician participants’ order of approach was randomized using computer-generated random numbers to avoid familiarity with the model for each subsequent approach. Therefore, the participants in each subgroup accomplished the approaches (transverse, longitudinal, or oblique) in six different orders.

**Figure 1:**
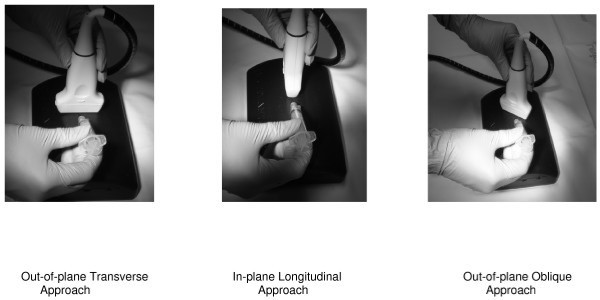
**The three approaches to vessel aspiration.** Out-of-plane Transverse Approach, In-plane Longitudinal Approach, Out-of-plane Oblique Approach.

**Figure 2:**
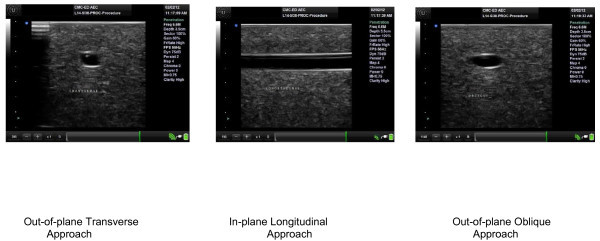
**Sonographic view of each approach.** Out-of-plane Transverse Approach, In-plane Longitudinal Approach, Out-of-plane Oblique Approach.

First, all participants were asked if they had ever performed any of the three techniques in the past, and if so, which was the most familiar. Next, the participants were given an approximately 5-min in-service training in vessel aspiration on the Blue Phantom Gel Model in each of the three approaches prior to their attempts. We used aspiration of the phantom vessel as a surrogate for venous access. Instructions included the use of the aforementioned high-frequency linear probe on the gel model. The out-of-plane transverse approach, the in-plane longitudinal, and the out-of-plane oblique approaches were demonstrated. More specifically, the transverse approach included the marker to the right of the gel model in a transverse axis at 90° to vessel direction. The longitudinal approach included the marker pointed toward the top (simulating the patients head) of the gel model at 0° to vessel direction. The out-of-plane oblique approach included the marker being between 0° and 90° to needle entry, pointing toward the right side of the gel model at 45°. Using an out-of-plane technique, the needle approaches the probe in a perpendicular fashion (refer to Figures 1 and 2). The participants were able to visualize needle entry on the ultrasound machine during the demonstration of each approach. No practice attempts were given.

The physician participants were informed to obviously retract their needle for each redirection or attempt to aspirate, just as would be done in a real patient, rather than digging through the gel. No other information or technical descriptions were offered prior to the attempts. Physician participants were not allowed to practice prior to the start of the study.

The start time was defined as the time when the needle touched the gel phantom model, not the time that the image was acquired by the participant. This was recorded by the study investigators using the same device each time. The total number of attempts to aspirate the vessel was also counted for each attempt by the research assistant. The first attempt was defined as penetration of the gel model, and each subsequent attempt was defined by needle retraction and forward movement, even if the needle did not leave the gel. Successful aspiration was defined as 1 cc of red aspiration fluid from the gel model seen in the syringe. The participant would be given no longer than 20 min total for all three approaches.

### Statistical analysis

Descriptive statistics including means and standard deviations or counts and percentages were calculated. Each subject was measured three times (once for each of the three approaches) for each of the outcome measures. Statistics that take this correlation or pairing into account were required for the analyses. For data measured on the interval scale (e.g., time to first success), a repeated measures analysis of variance was used. For data that was not normally distributed, Friedman’s nonparametric two-way analysis of variance was used, followed by the Wilcoxon signed rank tests when appropriate. For dichotomous data (success on the first attempt), the Cochran Q test was used. Since the difference between the first-attempt success rate of transverse, longitudinal, and oblique approaches is statistically significant by Cochran’s Q test, McNemar’s test was applied to compare each approach with another on a one-to-one basis. The *p* values for these three comparisons, as well as the three Wilcoxon signed rank tests, were adjusted by the Bonferroni correction. The outcomes were also compared among each of the four levels of experience using analysis of variance, the Kruskal-Wallis test, or a chi-square/Fisher’s exact test. The SAS software (SAS Institute, Cary, NC, USA) was used for all analyses. A two-tailed *p* value of less than 0.05 was considered statistically significant.

## Results and discussion

### Results

A total of 24 physicians participated with various training levels including PGY1, PGY2, PGY3, and attending physicians. All participants used the three approaches as described in the ‘Methods’ section. No participant took longer than approximately 3 min to locate the vessel under ultrasound and perform all of the three techniques.

The result of the initial survey was that no participant had ever attempted the oblique technique before. The most familiar and most widely used technique by all participants was the transverse approach. The highest success rates were demonstrated by the PGY3 and attending physician groups for all three approaches.

Table 1 shows the mean, median, maximum, and minimum scores for time to aspiration and number of attempts overall. The mean time to complete each approach was 9.47, 9.74, and 12.50 seconds for T, L, O approaches, respectively. When comparing the T, L, and O approaches for time, the *p* value was 0.572. The mean number of attempts were 1.75, 1.71, and 2.33 for the T, L, O approaches, respectively. See Table 2.

**Table 1:** Mean, median, minimum, and maximum aspiration times and number of attempts overall

**Variable**	**Mean**	**Standard deviation**	**Median**	**Minimum**	**Maximum**
Time					
Time T (s)	9.47	10.15	5.15	2.30	36.90
Time L (s)	9.74	14.44	5.25	1.00	63.10
Time O (s)	12.50	11.08	8.15	2.10	45.20
Attempt					
Attempt T	1.75	1.51	1.00	1.00	6.00
Attempt L	1.71	2.16	1.00	1.00	10.00
Attempt O	2.33	1.83	2.00	1.00	9.00

The *p* value for times when comparing T, L, and O is 0.572. When comparing attempts, the *p* value for Cochran’s Q was 0.046. The *p* values for the Wilcoxon signed ranked tests or the adjustment Bonferroni correction were not significant for the approaches when compared two at a time.

**Table 2:** Mean aspiration time and mean number of attempts by experience level

**Variable**	**PGY1 mean**	**PGY2 mean**	**PGY3 mean**	**Attending mean**	***p*value**
Time T (s)	10.43	16.98	6.12	4.33	0.131
Time L (s)	15.45	14.67	4.47	4.37	0.371
Time O (s)	17.50	15.45	6.22	10.83	0.007
Attempt T	2.17	2.67	1.17	1.00	0.131
Attempt L	2.17	2.50	1.00	1.17	0.530
Attempt O	3.67	2.50	1.33	1.83	0.132

When comparing the three approaches for attempts, the *p* value for Cochran’s Q was 0.046. Comparing two approaches at a time reveals *p* values of 0.230, 0.572, and 0.087 for T vs O, T vs L, and O vs L, respectively. However, the *p* values for the Wilcoxon signed rank tests were not significant. Adjusting with the Bonferroni Correction reveals *p* values of 0.690, 1.00, and 0.231 for T vs O, T vs L, and O vs L, respectively. The least and most amount of time to execute the technique was for the longitudinal approach at times of 1 and 63 s, respectively.

The first-attempt success rates at aspiration were 70.8%, 83.3%, and 45.8% by transverse, longitudinal, and oblique approaches, respectively. By McNemar’s test, first-attempt success rates between the longitudinal and oblique approaches were statistically significant (*p* = 0.03). The first-attempt success rates between the transverse and the oblique as well as the transverse and longitudinal approaches were not statistically significant (*p* = 0.09 and *p* = 1, respectively).

Reviewing the data among the subgroups reveals differences, although not statistically significant differences, when comparing the four experience levels. The PGY 3 and attending physicians took less time to aspirate and had fewer attempts on average (refer to Table 2).

### Discussion

With increasing frequency, ED physicians have sought to use ultrasound guidance for peripheral venous lines just as they have for central lines. Study results for peripheral line placement under ultrasound guidance have shown variable success. In 1999, Keyes et al. showed that cannulation of the brachial and basilic vein was successful in 91% of patients in their ED with difficult intravenous access [2]. In 2004, Brannam et al. showed that emergency nurses had a high success rate and few complications with the use of ultrasound guidance for peripheral vascular access after 45 min of training [3]. In 2005, Constantino et al. revealed a significant increase in success rate and overall less time for cannulation of peripheral veins under ultrasound guidance [4]. In 2007, Bauman et al. showed similar success rates for both traditional and ultrasound-guided approaches to peripheral access by ED technicians. Here, the ultrasound-guided group demonstrated improved speed and patient satisfaction with fewer skin punctures and complications [5]. Recently, however, Stein et al. showed that ultrasound-guided peripheral intravenous cannulation did not decrease the number of attempts or the time to successful catheterization [6]. In fact, this paper stated the time to cannulation was increased, a median of 13 min, in the ultrasound-guided group versus the group without ultrasound.

In all of the aforementioned studies cited in this paper thus far, the ultrasound-guided techniques for line placement utilized a longitudinal or transverse approach. The main drawback of the transverse approach is that the needle can deviate from the ultrasound beam and thus be lost from view [10]. When utilizing the longitudinal approach, it can be difficult to maintain probe placement in an exact location as to avoid ‘sliding off’ the structure outside its linear confines.

To avoid these pitfalls, many anesthesiologists prefer the out-of-plane oblique approach to venous cannulation [10]. The oblique approach may have the potential advantages of both the traditional approaches in terms of visualization. Gray states that with the out-of-plane approach, the target is typically centered within the field of view and the depth noted [10]. It has also been described scarcely in the emergency medicine literature as an alternative that allows visualization of the surrounding structures, entire needle, and needle tip [9].

This is the first prospective study we are aware of comparing simulated ultrasound-guided peripheral venous access using an oblique approach. We chose to compare a cohort with four subgroups of experience levels. At the start of each enrollment, we asked if the physician had performed the transverse, longitudinal, or oblique approach in the past. No participant had attempted an oblique approach before, and the most familiar technique was the transverse approach.

While it was expected that the transverse approach would be favored, the transverse and longitudinal approaches had similar rates of success. The oblique approach was less successful than the other two traditional approaches.

The shortest time to aspiration by the oblique and longitudinal approaches was 2.10 and 2.30 s, respectively. The transverse, which was most popular when all physician participants were initially surveyed, actually had the longest minimum time to aspiration overall. Among those who are highly skilled at ultrasound-guided vascular access, there was virtually no difference between aspiration times in all three approaches.

While PGY3 and attending physicians tended to aspirate in less time and by fewer attempts in all three orientations, these results did not reach statistical significance. Although statistically significant by Cochran’s Q (*p* = 0.046), the three approaches compared two at a time with the Bonferroni adjustment did not reveal statistical significance. In our study, experience level appeared to play a role in the success of peripheral venous aspiration, despite the approach used. It may have been that both the PGY3 and attending physician participants were more comfortable performing all three approaches than the PGY1 or PGY2 groups due to more familiarity with venous aspiration and cannulation in general at our institution. We would like to point out that higher education level may not always correlate to more experience in other departments where US is not utilized or a graduated training program in US is not in place.

### Limitations

Limitations of our study include the use of a simulated gel phantom, the limited amount of training for each technique, unfamiliarity with the technique and gel phantom by participants, and the study’s small sample size. The first limitation is that in this study, the phantom model was aspirated and not cannulated. The notion that successful aspiration directly corresponds to successful cannulation in an actual vessel would be incorrect. While an operator may be able to withdraw blood from a vein, it does not mean that they will actually be able to feed a catheter. In this study, we did not cannulate the phantom gel model so as not to destroy it. Second, we did not attempt to compare aspirating from the gel model with aspiration from a human vein. Rather, we chose to simply compare the three approaches on the same phantom model in this study. A third limitation is that the PGY1 participants, 24% of the total participants, had not used a gel phantom prior to enrollment, whereas 76% of the participants had. This may account for the longer times to aspiration for all three approaches when compared to the other participants. A fourth limitation is that the oblique technique was introduced but not practiced by any participant prior to enrollment. The oblique approach had been attempted by 0% of the participants prior to enrollment, whereas the other two traditional approaches had been performed by 100% of the participants prior to the study. It appears that unfamiliarity with the oblique approach is the main contributing factor for its poorer performance overall. However, it is noteworthy that the participants who have been practicing medicine and ultrasound guidance longer (the PGY3 and attending participants) did have a less difficult time with the oblique than the others in our study. Another study that includes practice time for the oblique approach prior to aspiration would better compare the three approaches among all participants in the future. Lastly, our study had a small sample size. A larger study would be necessary to confirm our results.

## Conclusion

In a pilot study of simulated ultrasound-guided peripheral venous access by emergency physicians, the out-of-plane oblique approach did not reduce the time to aspiration or total number of attempts. Our study suggested that higher educational level improved success, regardless of approach. Further studies using both the in-plane and out-of-plane oblique approaches for ultrasound-guided peripheral venous access should be performed to fully compare the oblique approach to the traditional transverse and longitudinal techniques.

## Competing interests

The authors declare that they have no competing interests.

## Authors’ contributions

HT and VT conceived the design of the study. HT, AW, and CG enrolled each participant and coordinated the study. JN participated in the design and performed the statistical analysis. HT drafted the manuscript. HT and VT provided images for the manuscript. HT and VT revised and edited the manuscript. All authors read and approved the manuscript.
